# Ostruthin, a TWIK-Related Potassium Channel Agonist, Increases the Body Temperature in Ovariectomized Rats With or Without Progesterone Administration

**DOI:** 10.7759/cureus.65706

**Published:** 2024-07-29

**Authors:** Yuki Uchida, Yuki Samejima, Shotaro Kamijo, Masahiro Hosonuma, Masahiko Izumizaki

**Affiliations:** 1 Department of Physiology, Showa University School of Medicine, Shinagawaku, JPN; 2 Department of Orthopedic Surgery, Showa University Fujigaoka Hospital, Yokohama, JPN; 3 Division of Physiology, Toxicology and Therapeutics, Department of Pharmacology, Showa University School of Medicine, Shinagawaku, JPN; 4 Division of Medical Pharmacology, Department of Pharmacology, Showa University School of Medicine, Shinagawaku, JPN

**Keywords:** locomotor activity, tail skin temperature, body temperature, twik-related potassium channels, ostruthin

## Abstract

Background and objectives: The TWIK-related potassium (TREK) channel subfamily, including TREK1 and TREK2, is a novel cold receptor. Ostruthin, a TREK1 and TREK2 agonist, is a component found in the plant *Paramignya trimera* and is traditionally used as an anticancer medicine in Vietnam, with its stems and roots treating various ailments. The female hormone progesterone (P4) influences body temperature in women; however, the effect of P4 on thermoregulation via TREK has not been examined. This study aims to investigate the effects of P4 on thermoregulatory responses in ostruthin-administered ovariectomized rats, which are animal models of human menopause.

Methods: Wistar rats were ovariectomized and implanted with silastic tubes with or without P4 (P4(+) and P4(-) groups). The TREK agonist or vehicle was injected intraperitoneally. Body temperature, locomotor activity, tail skin temperature, and thermoregulatory behavior (assessed by tail-hiding behavior) were continuously measured. Plasma concentrations of catecholamines, triiodothyronine, and thyroxine were also measured.

Results: In both the P4(+) and P4(−) groups, the change in body temperature was greater among the rats administered the TREK agonist compared to the vehicle. No significant differences were observed between the groups in locomotor activity, tail skin temperature, or tail-hiding behavior. The dopamine concentration in the P4(+) group was lower than that in the P4(-) group.

Conclusions: Ostruthin, the TREK agonist, increases body temperature in ovariectomized rats; however, P4 may not affect these responses in ovariectomized rats.

## Introduction

Maintaining body temperature (T_b_) through both autonomic and behavioral thermoregulation in cold environments is essential for the survival of animals and humans [1]. Thermoregulation is classified into autonomic (e.g., vasoconstriction, shivering, and non-shivering thermogenesis) and behavioral thermoregulation (e.g., cold escape behavior) [1]. These thermoregulatory processes are influenced by various factors, including sex hormones [1]. The decline in female hormones associated with menopause often causes thermoregulatory problems, such as hot flashes [2]. These symptoms are thought to result from decreased estrogen (E_2_) and progesterone (P4) affecting thermoregulation [2].

Our previous research demonstrated that E_2_ enhances both autonomic and behavioral thermoregulation in ovariectomized rats exposed to cold environments, serving as an animal model for human menopause [3]. However, the effect of P4 on autonomic and behavioral thermoregulation through peripheral cold receptors in ovariectomized rats remains unexamined [4]. This study aims to examine the effect of P4 on T_b_ through new cold receptors, TWIK-related potassium channels (TREK), in ovariectomized rats.

Cold receptors play a crucial role in triggering autonomic and behavioral thermoregulation. TREK1 and TREK2 presented in skin sensory neurons are subtypes of TREK [5]. In vitro studies show TREK1 activation at 14-40°C [6] and TREK2 at 40-46°C and 20°C-25°C in mice dorsal root ganglia [7]. Additionally, TREK2-/- mice showed a decrease in tail immersion test latency at mild cold temperatures [7]. These results suggest that TREK1 and TREK2 are crucial mild cold receptors.

Systemic [8] and local administration [9] of P4 to the preoptic area of the hypothalamus increased T_b_ in ovariectomized rats at normal ambient temperature. Thus, we hypothesized that activation of TREK1 and TREK2 would increase T_b_ by mimicking cold stimulation, and P4 administration would enhance this effect in ovariectomized rats.

To test our hypotheses, we used ostruthin, a plant-derived compound known to activate TREK1 and TREK2, as an agonist [10]. We assessed various thermoregulatory parameters in ovariectomized rats, including a novel indicator of behavioral thermoregulation: tail-hiding behavior [11]. This approach allows us to explore the potential role of TREK in P4-mediated thermoregulation, offering a new perspective on the interactions of female hormones and T_b_ in cold environments in menopausal women.

## Materials and methods

Animals

Virgin female Wistar rats (n = 32; body weight, 165 ± 0.3 g; age, 9 weeks; Japan SLC, Hamamatsu, Japan) were used in this study. The rats were individually housed in cages (37 × 21 × 19 cm) at an ambient temperature of 23 ± 1°C in a 12:12-hour light-dark cycle (lights on at 08:00 h) and had free access to food and water before the experiment. The Animal Research Committee of Showa University (Tokyo, Japan) approved all experimental protocols (approval number: 03110).

Surgery

The rats underwent surgery under isoflurane inhalation anesthesia (Pfizer Japan, Tokyo, Japan). Following a medial skin incision, a sensor with a built-in data logger (nano tag®, Kissei Comtec Co. Ltd., Nagano, Japan) was implanted into the peritoneal cavity of each rat to measure body temperature (T_b_) and locomotor activity (Act) for thermogenesis by muscle activation. The sensor had a temperature accuracy of approximately 0.0625°C. Locomotor activity was detected using a triaxial accelerometer. Bilateral ovariectomy was performed through a dorsal skin incision. To prevent postoperative infection, the animals were injected subcutaneously with penicillin G (1,000 U; Meiji Pharmaceutical, Tokyo, Japan). The ovariectomy method followed was as described in our previous study. After ovariectomy, plasma estradiol and progesterone concentrations reached low levels, as shown in Figure 3C and our previous study [12].

Experimental protocols

One week after ovariectomy, the rats were divided into four groups: Vehicle/P4(-), Vehicle/P4(+), TREK agonist/P4(-), and TREK agonist/P4(+). One day before the experiment, the rats were implanted with two silastic tubes (Dow Corning Toray Co., Ltd, Tokyo, Japan; OD, 3.18 mm; ID, 1.57 mm) beneath the right dorsal skin under isoflurane anesthesia. The inserted tube was 30 mm long, with 5-mm wooden sticks inserted at the end of the tube (effective capsule length, 20 mm). Tubes containing P4 (P4(+); 50 mg/mL in sesame oil; FUJIFILM Wako Pure Chemical Corporation, Osaka, Japan; n = 16) or sesame oil (P4(-); n = 16) were implanted. The implanted P4 tubes maintained pharmacological levels in plasma (Figure 3C-a). The method of P4 tube implantation has been described in a previous study [13].

On the experiment day, each rat in the groups was transferred to a polyethylene box (19.8 × 30.3 × 48.5 cm) and moved to a climatic chamber (Program Incubator IN804, Yamato Scientific, Tokyo, Japan) kept at 27°C for 2 hours (08:00-10:00). At 10:00, the rat was administered an intraperitoneal (ip) vehicle (Vehicle/P4(-) and Vehicle/P4(+) groups; 0.002% dimethyl sulfoxide (DMSO; FUJIFILM Wako Pure Chemical Corporation) in phosphate-buffered saline; 200 μL) or TREK1 and TREK2 agonists (TREK agonist/P4(-) and TREK agonist/P4(+) groups; ostruthin (Toronto Research Chemicals, Toronto, ON, Canada) 4.2 μg/0.002% dimethyl sulfoxide in phosphate-buffered saline; 200 μL) based on a previous study [10] and our preliminary experiment. After the intraperitoneal administration, the rat was moved to a polyethylene box and exposed to 27°C for 2 hours (10:00-12:00), according to our previous study [14]. The rats were not provided food or water during the experiment.

We used ostruthin as a TREK1 and TREK2 agonist. Ostruthin, a plant component found in *Paramignya trimera*, is traditionally used as an anticancer medicine in Vietnam. Paramignya trimera, also known as Xáo tam phân, is common in southern Vietnam and its stems and roots are used to treat various ailments [15]. Electrophysiological studies have shown that ostruthin activates TREK1 and TREK2. In vivo, ostruthin administration exhibited antidepressant effects in forced swim and tail suspension tests in mice, and reduced stress-induced neural activity in the lateral septum [10]. These findings indicate that ostruthin induces physiological responses by activating TREK1 and TREK2 in vivo.

Infrared thermography (Thermo Gear G100, Nippon Avionics Co., Ltd., Kanagawa, Japan) was used to monitor tail skin temperature (T_tail_) for heat dissipation and tail-hiding behavior of the rat at one-minute intervals. The behavior was counted as tail-hiding if the rat hid its entire tail under its body, as previously described [11]. Infrared thermography was placed 90 cm above the rat and showed an accuracy of ±0.06°C. Based on the thermograms, T_tail_ was analyzed at two points (one-third of the tail length from the tail tip and root) and averaged. When the tail could not be observed, the missing T_tail_ value was replaced with a value observed at a previously recorded time point, as previously described [14]. The T_tail_ value was calibrated using a compensation formula obtained from a calibration experiment using a thermocouple. The total duration of tail-hiding behavior was observed using thermograms after intraperitoneal administration.

Various artificial systems have been used to study behavioral thermoregulation in cold environments, such as operant behavior tests and thermal gradient systems [16]. However, these methods require pre-training of rodents and do not reflect natural thermoregulatory behavior. Additionally, the temperature of thermal gradient systems affects T_b_ in animals. Therefore, in this study, we used tail-hiding behavior, a newly defined thermoregulatory behavior in rats under cold conditions that we discovered [11].

After the measurements, the rats were sacrificed by isoflurane inhalation anesthesia. A 2-mL blood sample was taken from the left ventricular cavity and collected in tubes containing EDTA-2Na, according to our previous study.

Evaluation of Plasma Catecholamine, Triiodothyronine, Thyroxine, P4, and E_2_ Levels

Plasma levels of catecholamines were measured to evaluate sympathetic activity related to thermoregulation [17], and thyroid hormone levels were assessed for thermogenesis [18]. The collected blood was centrifuged at 4°C, and the plasma was stored at −80°C until the assay. Plasma levels of catecholamines (CA test TOSOH; Tosoh Corporation, Tokyo, Japan; HPLC system; Shimadzu Corporation, Kyoto, Japan; Hitachi, Ltd., Tokyo, Japan; Jasco Corporation, Tokyo, Japan; HPLC), triiodothyronine and thyroxine (Lumipulse Presto® T4; Fujirebio Inc., Tokyo, Japan; fully automatic CLEIA system Lumipulse L2400; Fujirebio Inc., Tokyo, Japan; CLEIA), progesterone (P4; Elecsys Progesterone III; Roche Diagnostics KK, Tokyo, Japan; Cobas 8000 e801 analytical unit, Hitachi High-Tech Corporation, Tokyo, Japan; ECLIA) and estradiol (E_2_; Elecsys Estradiol IV; Roche Diagnostics KK, Tokyo, Japan; Cobas 8000 e801 analytical unit, Hitachi High-Tech Corporation, Tokyo, Japan; ECLIA) were determined at SRL Inc. (Tokyo, Japan). All measurements were performed according to the manufacturer's instructions.

Statistics

Data are presented as mean ± standard error. The changes in T_b_ (∆T_b_), Act (∆Act), and T_tail_ (∆T_tail_) were calculated from the baseline values of T_b_, Act, and T_tail_ (the mean for the 30 min before the injection of vehicle or TREK agonist) based on our study [14]. The values for ∆T_b_, ∆Act, ∆T_tail_, and the heat loss index were averaged over a period of 30 min.

Differences in ∆T_b_, ∆Act, and ∆T_tail_, and the heat loss index between P4(-) and P4(+), as well as between vehicle and TREK agonist, were assessed using covariance analysis (ANCOVA). The covariate for analysis of variance (ANCOVA) was time. Differences in the duration and onset of tail-hiding behavior, plasma catecholamine, T3, T4, P4, and E_2_ levels between the P4(-) and P4(+) groups and between the vehicle and TREK agonist groups were assessed using two-way ANOVA. The post-hoc Tukey honestly significant difference test for multiple comparisons was performed. Statistical analyses were performed using the IBM SPSS Statistics for Windows, Version 21 (Released 2012; IBM Corp., Armonk, New York). Statistical significance was set at p < 0.05.

## Results

**Figure 1:**
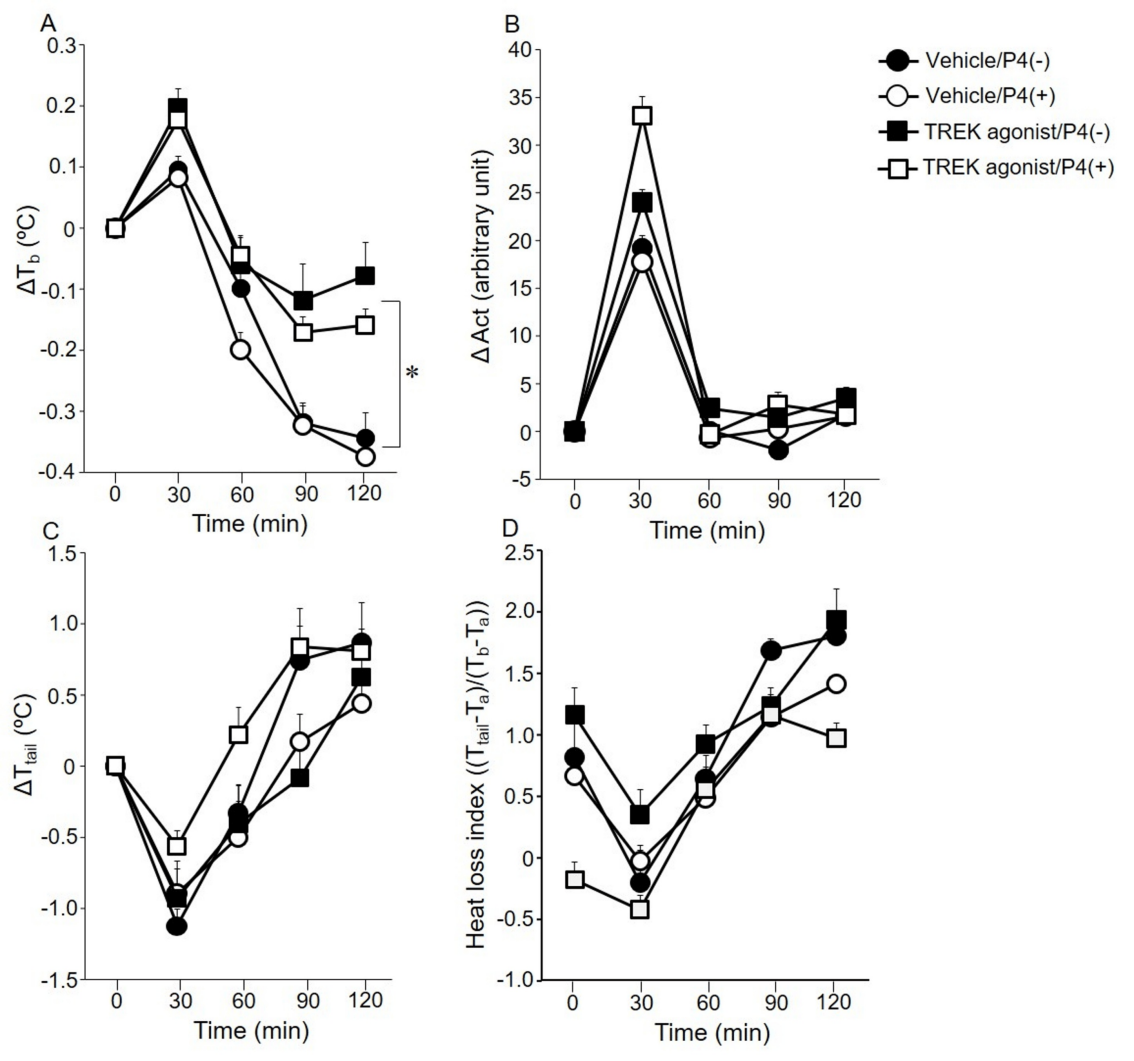
Change in body temperature (A), locomotor activity (Act) (B), tail skin temperature from the baseline (C), and heat loss index (D). Values are presented as mean ± standard error (n=8/group). ∆T_b_, ∆Act, ∆T_tail_, and heat loss index were assessed using covariance analysis. The covariate for the covariance analysis was time. The post-hoc Tukey honestly significant difference test for multiple comparisons was performed. Significant differences between vehicle and TWIK-related potassium channels (TREK) agonist (*), p<0.05. P4: progesterone.

Figure 1 shows ∆T_b_ (A), ΔAct (B), ΔT_tail _(C), and the heat loss index (D). The baseline of T_b_ did not differ between the groups (Vehicle/P4(-), 37.0 ± 0.0°C; Vehicle/P4(+), 37.1 ± 0.0°C; TREK agonist/P4(-), 36.8 ± 0.0°C; TREK agonist/P4(+), 36.9 ± 0.0°C). The ANCOVA results indicated a significant main effect of TREK agonist on ∆T_b_ (F(1,123) = 9.37, p < 0.05), with higher ∆T_b_ observed in the TREK agonist/P4(-) and TREK agonist/P4(+) groups. The baseline of the Act did not differ between the groups (Vehicle/P4(-), 3 ± 0; Vehicle/P4(+), 3 ± 0; TREK agonist/P4(-), 2 ± 0; TREK agonist/P4(+), 3 ± 0). The baseline of T_tail_ did not differ between the groups (Vehicle/P4(-), 28.6 ± 0.3°C; Vehicle/P4(+), 28.7 ± 0.2°C; TREK agonist/P4(-), 29.1 ± 0.2°C; TREK agonist/P4(+), 28.0 ± 0.2°C). No significant differences were observed in ΔAct, ΔT_tail,_ and the heat loss index between any of the groups.

**Figure 2:**
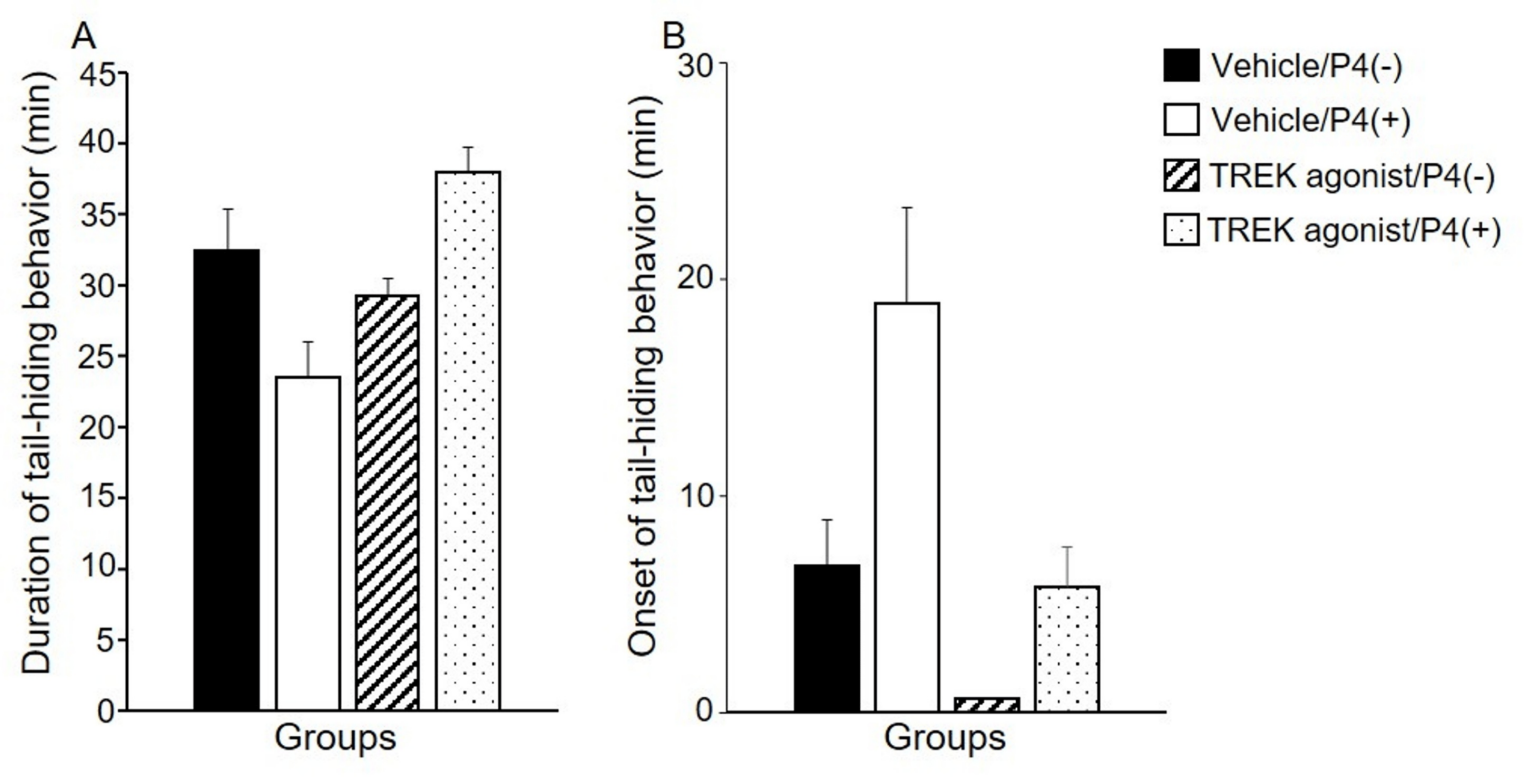
Duration (A) and onset (B) of tail-hiding behavior. Values are presented as mean ±standard error (n=8/group). The duration and onset of tail-hiding behavior were assessed using a two-way analysis of variance. TREK: TWIK-related potassium channels, P4: progesterone.

Figure 2 shows the duration (A) and onset (B) of tail-hiding behavior. No significant differences were observed between the groups in the duration or onset of tail-hiding behavior.

**Figure 3:**
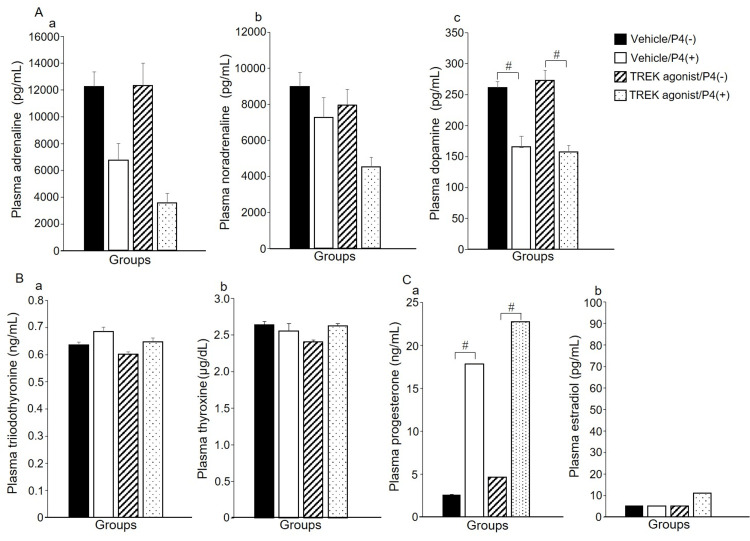
Plasma levels of adrenaline (A-a), noradrenaline (A-b), dopamine (A-c), triiodothyronine (B-a), thyroxine (B-b), P4 (C-a), and E2 (C-b) concentrations. Values are presented as mean ± standard error (adrenaline, noradrenaline, and dopamine (Vehicle/P4(–) and TREK agonist/P4(–) groups, n=8; Vehicle/P4(+) and TREK agonist/P4(+) groups, n=7), triiodothyronine and thyroxine (Vehicle/P4(–) group, n=8; Vehicle/P4(+) group, n=5; TREK agonist/P4(–) and TREK agonist/P4(+) groups, n=7), P4 and E2 (Vehicle/P4(–) and TREK agonist/P4(–) groups, n=8; Vehicle/P4(+) group, n=5; TREK agonist/P4(+) group, n=7). All values were assessed using a two-way analysis of variance. The post-hoc Tukey honestly significant difference test for multiple comparisons was performed. Significant differences between P4(–) and P4(+) (#), p<0.05. TWIK-related potassium channels, TREK; P4, progesterone.

Figure 3 shows plasma levels of adrenaline (A-a), noradrenaline (A-b), dopamine (A-c), triiodothyronine (B-a), thyroxine (B-b), P4 (C-a), and E_2_ (C-b) concentrations. No significant differences were observed in plasma adrenaline, noradrenaline, triiodothyronine, thyroxine, or E_2_ levels between the groups. The two-way ANOVA indicated a significant main effect of P4 on dopamine concentration (F(1,26) = 7.52, p < 0.05). Dopamine concentrations in the Vehicle/P4(+) and TREK agonist/P4(+) groups were lower than those in the Vehicle/P4(-) and TREK agonist/P4(-) groups (p < 0.05). Two-way ANOVA indicated a significant main effect influenced by P4 concentration (F(1,25) = 49.71, p < 0.05). P4 concentrations in the Vehicle/P4(+) and TREK agonist/P4(+) groups were higher than those in the Vehicle/P4(-) and TREK agonist/P4(-) groups (p < 0.05).

## Discussion

The present study revealed that P4 did not influence thermoregulatory responses induced by the TREK agonist in ovariectomized rats. However, the TREK agonist prevented the decrease in T_b_ in ovariectomized rats, regardless of P4 presence.

First, we verified the method of P4 administration in ovariectomized rats by checking plasma P4 levels across all groups. Plasma P4 concentrations in the proestrus and on the first day of diestrus in the estrous cycle of female rats were approximately 5 ng/mL, indicating pharmacological levels. In our previous study, the plasma P4 concentration in ovariectomized rats was approximately 2.5 ng/mL [12]. Plasma P4 concentration in the P(-) group matched these levels. Furthermore, plasma E_2_ concentration in ovariectomized rats was 25 pg/mL [12]. The E_2_ concentration in the P(-) group was lower than that reported in our previous study [12]. Hence, the ovariectomy method in this study effectively decreased female hormone levels. These findings confirm the appropriateness of the ovariectomy and P4 administration methods used.

We examined the effect of P4 on T_b_ in ovariectomized rats with or without the TREK agonist. At normal ambient temperature, systemic [8] and local administration of P4 to the preoptic area of the hypothalamus [9] increased T_b_ in the ovariectomized rat. Conversely, systemic administration of P4 (10 mg/21 days) decreased T_b_ in ovariectomized mice [19]. These results suggest that the effect of a single administration of P4 on T_b_ in ovariectomized rats is controversial. Our results neither aligned with these previous findings nor supported our hypothesis. The effect of P4 on T_b_ in ovariectomized rats may depend on the dose and duration of P4 administration. Our study suggests that P4 does not affect T_b_ in ovariectomized rats, regardless of TREK agonist administration. Conversely, the TREK agonist mitigated the reduction in T_b_ in ovariectomized rats irrespective of P4 presence.

Our findings on Act align with a study where subcutaneous administration of P4 (500 μg) did not affect locomotor activity in ovariectomized rats [20]. The intraperitoneal administration of the TREK agonist (2, 5, and 20 mg/kg) did not influence the locomotor activity of mice, as assessed by running on a wheel [10]. Thus, neither the P4 nor TREK agonist affects thermogenesis through Act. Our results on T_tail_ are consistent with the findings of previous studies in which subcutaneous administration of P4 (0.3 and 1.5 mg/d) did not affect T_tail_ in ovariectomized rats [21]. In addition, this study demonstrated that neither the TREK agonist nor P4 influenced thermoregulatory behavior assessed by tail-hiding behavior. Therefore, the suppression of the reduction in T_b_ by TREK agonist is not due to heat dissipation by T_tail_, tail-hiding behavior, or thermogenesis by Act.

Our findings in plasma catecholamine levels are partially aligned with previous studies showing that subcutaneous administration of P4 (50 mg, 21 days) did not influence plasma adrenaline and noradrenaline levels in ovariectomized rats [22]. Our results partially coincide with previous reports indicating that subcutaneous administration of P4 (0.5 mg/100 g, 6 days) reduces plasma dopamine levels in rats [23]. Dopamine decreases the rectal temperature in rats [24]. Therefore, P4 may inhibit the reduction of T_b_ by dopamine; however, P4 did not affect T_b_. The reduction in dopamine caused by P4 might not be sufficient to affect T_b_. Systemic sympathetic nerve activity, assessed using plasma catecholamines, did not contribute to the suppression of the decrease in T_b_ by the TREK agonist.

Administration of P4 (50 mg/mL once daily for three days) increased plasma thyroxine levels in ovariectomized rats [25]. In contrast, another study reported that intramuscular injection of P4 (1 mg/100 g, 30 days) decreased plasma thyroxine levels but increased triiodothyronine levels in ovariectomized rats [26]. Our results differ from previous findings, suggesting that variations in dose, duration (short vs. long), and administration method (subcutaneous vs. intramuscular) lead to differences in plasma triiodothyronine and thyroxine levels. Therefore, thyroid hormone-induced thermogenesis does not account for the suppression of the reduction in T_b_ by the TREK agonist in ovariectomized rats.

Brown adipose tissue is the main organ of non-shivering thermogenesis in rats and is activated by sympathetic nerve activity [27]. Direct activation of cold receptors, such as transient receptor potential cation channel subfamily M member 8 (TRPM8), expressed in brown adipose tissue by menthol, the TRPM8 agonist, induces metabolism and thermogenesis in mice [28]. While the expression of TREK channels in brown adipose tissue has not been reported, TREK1 mRNA and transcript were found in mesenchymal stromal cells of human adipose tissue in vitro [29]. Therefore, it is possible that the TREK agonist directly affects TREK1 expressed in brown adipose tissue, facilitating non-shivering thermogenesis. Considering all the results, the TREK agonist might mitigate the reduction in T_b_ by inducing thermogenesis through TREK1 in brown adipose tissue. Future studies should evaluate the effect of the TREK agonist on thermogenesis in brown adipose tissue in ovariectomized rats.

In terms of clinical implications, these results may be related to "hie-sho" and hypothermia in menopausal women. Japanese menopausal women who feel cold, even in a warm room, are said to experience ‘hie-sho’ [30]. The administration of the TREK agonist may help improve hie-sho and hypothermia in both pre- and postmenopausal women, as it suppresses the decrease in T_b_ in ovariectomized rats with or without the presence of P4.

This study has several limitations. We did not include sham and intact groups, as our aim was to examine the effect of a single P4 administration on T_b_ in ovariectomized rats administered the TREK agonist. Additionally, the effect of the TREK agonist on Tb and thermoregulatory behavior in intact animals has not been examined in previous studies. P4 interacts with and antagonizes E2. Future studies should include the co-administration of P4 and E2 to examine Tb via TREK in vivo. Additionally, rats were used instead of mice to measure tail-hiding behavior, as the physiological function of tail-hiding behavior in mice has not been studied. P4 binds to both P4 and steroid hormone receptors. Future research should use P4 and steroid hormone receptor knockout mice to investigate the effect of P4 on thermoregulation via these receptors in knockout mice administered the TREK agonist. Furthermore, if specific agonists for TREK1 and TREK2 suitable for in vivo studies are developed, they should be used, although ostruthin was used as a TREK agonist in this study.

## Conclusions

In conclusion, the present study revealed that P4 did not affect thermoregulatory responses induced by the TREK agonist. However, administration of the TREK agonist suppressed the decrease in T_b_ in ovariectomized rats, regardless of P4 presence.
